# Flu and COVID-19 Vaccination: What Happens to the Flu Shot When the Campaigns Overlap? Experience from a Large Italian Research Hospital

**DOI:** 10.3390/vaccines10060976

**Published:** 2022-06-19

**Authors:** Domenico Pascucci, Mario Cesare Nurchis, Alberto Lontano, Eleonora Marziali, Giuseppe Vetrugno, Andrea Cambieri, Umberto Moscato, Andrea Di Pilla, Gianfranco Damiani, Patrizia Laurenti

**Affiliations:** 1 Università Cattolica del Sacro Cuore, 00168 Rome, Italy; domenico.pascucci@outlook.it (D.P.); nurchismario@gmail.com (M.C.N.); alberto.lontano@gmail.com (A.L.); giuseppe.vetrugno@unicatt.it (G.V.); umberto.moscato@unicatt.it (U.M.); andrea.dipilla01@icatt.it (A.D.P.); gianfranco.damiani@unicatt.it (G.D.); patrizia.laurenti@unicatt.it (P.L.); 2 Fondazione Policlinico Universitario A. Gemelli IRCCS, 00168 Rome, Italy; andrea.cambieri@policlinicogemelli.it

**Keywords:** flu vaccination, COVID-19, healthcare workers, vaccination coverage, public health

## Abstract

Influenza represents a threat to global health and health care workers (HCWs) have an increased risk of contracting the influenza virus in the workplace. The COVID-19 pandemic has brought back the importance of influenza vaccination, as the influenza virus can circulate together with SARS-CoV-2. The aim of this report is to describe the actual flu vaccination coverage among healthcare workers of a research hospital and the trend changes, with respect to the past flu vaccination campaigns, in light of the present pandemic and COVID-19 vaccination. A Pearson’s χ^2^ test was used to test the correlation of flu vaccination coverage, across all professional categories, between the last two years. A linear regression model was adopted to predict the total vaccination coverage of this year. A statistically significant decrease (*p* < 0.01) was observed in vaccination coverage among all the professional categories with a 50% reduction in vaccination trends between the last two years. Analyzing the data from the previous six flu vaccination campaigns, the expected value, according to the linear regression model, was estimated to be 38.5% while the observed value was 24%. The decrease in vaccination coverage may be due to the fear of the pandemic situation and especially to the uncertainty related to the consequences of a concurrent administration which may overload the immune system or may be more reactogenic. The COVID-19 pandemic represents an opportunity to promote and support large-scale influenza vaccination among HCWs through structured programs, adequate funding, and tailored communication strategies.

## 1. Introduction

Influenza represents a threat to global health in terms of mortality and morbidity. It is estimated that it can infect up to one billion individuals each year although severe cases are 3–5 million, with a death toll ranging from 290,000 to 650,000 [1].

Health care workers have an increased risk of contracting the influenza virus in the workplace, both from patients and from infected colleagues: they are at increased risk compared with the average healthy adult [2,3], and it is estimated that one in four healthcare workers may become infected during a mild flu season [2].

For this reason, the World Health Organization (WHO) considers healthcare workers a priority high-risk group for receiving influenza vaccination to protect themselves and their patients, and to ensure continuity of healthcare services [4].

The perception of risk towards influenza among healthcare workers varies according to the work setting (i.e., hospitals with high intensity of care or community hospitals) [5,6] and the care task performed: medical doctors show a greater acceptance of influenza vaccination, while nurses, midwives, nursing assistants, and nursing aides are less inclined to vaccination [5,7].

In addition, healthcare professionals employed in high-risk departments are more willing to be vaccinated, a phenomenon probably attributable to a greater perception of risk [8,9,10].

Although the optimal vaccination rate among health care professionals is 100%, a minimum rate of 80% is considered sufficient to prevent circulation of the virus [11,12].

However, healthcare professionals’ compliance with influenza vaccination and, consequently, vaccination coverages, are worryingly low [13].

For three consecutive vaccination seasons from 2015 to 2018, vaccination coverages among healthcare workers in 12 European Union states decreased, ranging from a minimum of 15.6% to a maximum of 63.2% [14]. These data are corroborated by several other studies, which have shown minimum coverages of even less than 10% [5,15,16].

In 2017, 119 countries and regions used flu vaccine, and 102 countries had policies in place to vaccinate health care workers [10,14,17].

Significantly low coverages are common among medical residents that begin to be part of the HCW risk group when they start their internships in medical-care facilities [18,19].

Only 18.2% of Italian medical residents underwent influenza vaccination in the 2016–2017 season, far from the coverages achieved in the United States [20] and France [21] in previous vaccination seasons and from the goals of the national vaccine prevention plan [22]. These coverages are similar or even lower than those reported among HCWs [18,19,23,24].

The main reasons for this low adherence are a poor perception of the risk of becoming infected, the belief that patients will still develop symptoms despite vaccination, a lack of awareness of being able to transmit the infection to their patients, a lack of time due to very long shifts, and doubts about their efficacy [25,26,27].

Medical residents in the medical area seem to be more inclined to vaccination, perhaps because of the type of patients they take care of [25,26].

Several strategies have been implemented to increase healthcare workers’ adherence to influenza vaccination [28]. The most effective has proven to be compulsory vaccination, especially for healthcare workers in contact with high-risk patients [29,30]; however, this measure burdens workers’ morale and has non-negligible ethical and legal implications [28,31,32].

The effectiveness of other strategies is proportional to the number of them employed, and includes the use of free vaccination, reminders, vaccination in the workplace, and educational material tailored to different classes of health workers [28].

The COVID-19 pandemic has brought back the importance of influenza vaccination [33], as the influenza virus can circulate together with SARS-CoV-2 [34,35].

Both viruses have a high societal impact in terms of mortality and morbidity [36,37,38], and since both are respiratory viruses with similar symptoms, differential diagnosis is complex [35].

Several studies have shown that the COVID-19 pandemic has influenced the attitude and perception of HCWs to influenza vaccination [36,39,40].

In this regard, Stöckeler et al. [39] report a rise in the influenza vaccination coverage from 31% in the 2016/2017 season to 59% in the 2020/2021 season; similarly, Wang et al. outline a more positive attitude towards influenza vaccination, possibly due to a raised awareness of the risks related to airborne infections [41].

Moreover, Di Pumpo et al. report an increase in vaccine coverage in some professional classes not directly involved in taking care of frail patients, such as administrative or auxiliary staff, suggesting a correlation between the fear of COVID-19 and the increase in vaccination rates [42].

The aim of this report is to describe the actual flu vaccination coverage among healthcare workers of a research hospital and the variations, over the past six flu vaccination campaigns, in light of the present pandemic and COVID-19 vaccination.

## 2. Materials and Methods

A retrospective cohort study was conducted to evaluate the flu vaccination coverage among the health workers of the Fondazione Policlinico Universitario Agostino Gemelli IRCCS, an Italian high complexity research hospital. All personnel were offered vaccination by the hospital hygiene unit from October 2021 to January 2022. The personnel working at hospital units who could give written informed consent were included. The study protocol was approved by the research hospital Ethical Board with the approval number 10755/22 ID 3706.

The vaccines were provided free of charge to the HCWs by the Local Health Unit according to the Italian “Piano Nazionale Prevenzione Vaccinale” [22]. Two vaccine hubs were set up in different areas of our research hospital to favor a major accessibility from the HCWs. The vaccine hubs were run by specialist medical doctors in hygiene and preventive medicine, occupational medicine, resident medical doctors of the same areas, and nurses who delivered vaccination.

Administrative hospital databases were queried to retrieve vaccination data for the assessed time horizon (i.e., from October 2021 to January 2022). A database was set up defining firstly a unique anonymous identifier code for each individual with the specification of the professional occupation. The individual employee tax code was used to obtain socio-demographic data as the age and sex whereas information about occupation was acquired from the research hospital human resources unit.

In relation to the vaccine coverage analysis, the findings were preliminary described through frequencies and percentages. A Pearson’s χ^2^ test was used to test the correlation of flu vaccination coverage, across all professional categories, between the last two years.

Furthermore, a multivariable logistic regression was performed to investigate the influence of gender, age, and occupation on the likelihood towards vaccination.

Based on the assumption of the model, the dependent variable was dichotomized into health workers who got vaccinated and those who did not. The Hosmer–Lemeshow (HL) goodness of fit test was run to test how well the data fits the logistic model [43]. A significance higher than 0.05 showed a good model fitness. Moreover, a receiver operating characteristic (ROC) curve was designed to analyze the area under this curve (AUC) to assess the overall measure of model fit [44].

In addition, a linear regression model was adopted to predict the total vaccination coverage of this year [45,46]. Five per cent was the statistical level chosen for all the statistical analyses which were run on STATA 17 (StataCorp LP, College Station, TX, USA).

## 3. Results

Between October 2021 and January 2022, 8221 hospital workers were observed. A total of 1979 individuals were vaccinated, while 6242 were unvaccinated. Of the observed hospital workers, 40% were male, 47% were older than 40 years old, and 42% were medical doctors. The median age at vaccination was 39.

Table 1 depicts a statistically significant decrease in vaccination coverage among all the professional categories with, approximately, a 50% reduction in vaccination trends between the last two years.

The findings of the multivariate logistic regression showed that being a nurse was statistically significant associated with a lower vaccination uptake in comparison with being a physician (OR 2.35, 95% CI 2.02–2.73) or a resident medical doctor (OR 1.38, 95% CI 1.18–1.61). Moreover, the results highlighted that being aged between 40 and 59 (OR 1.26, 95% CI 1.11–1.43), compared with the youngest age class (i.e., between 20 and 39), was statistically significant associated with a higher uptake to vaccination. Interestingly, being male, is associated with a statistically significant reduction (OR 0.89, 95% CI 0.79–0.98) in vaccination uptake with respect to be female. According to the HL test, the model fitted quite well (*p* > 0.2). This result is also confirmed by AUC highlighting a good fit of the model (0.59, 95% CI 0.57–0.61).

Furthermore, analyzing the data from the previous six flu vaccination campaigns, the expected value, according to the linear regression model, was estimated to be 38.5%. However, the observed value was sharply lower, although not statistically significant, amounting to 24.0%.

**Figure 1:**
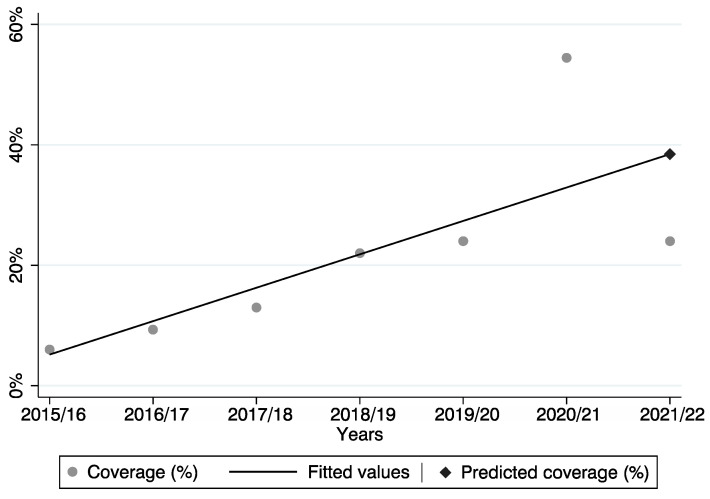
Predicted and observed vaccination coverage across the six vaccination campaigns.

Considering the data point of 20/21 (i.e., 54.6%) as an outlier, the coverage of the current vaccination campaign is similar to the one of the 19/20 campaign (Figure 1).

## 4. Discussion and Conclusions

Study findings show a major decline in the coverage achieved by influenza vaccination compared to the change of increasing coverage that can be described from 2016 onwards. The drop in coverage is even more pronounced when comparing with coverage for the 2020–2021 vaccination season, in which the absence of a SARS-CoV-2 vaccine prompted more healthcare workers to be vaccinated.

The results are in line with those reported by Public Health England, which found a decrease in influenza vaccination coverage among healthcare workers (76.8% in 2021 [47] vs. 60.5% in 2022 [48]), albeit less marked than that described in our study.

This trend, similar to that found in our study, was described for medical doctors (78.2% in 2020–2021 vs. 63.5% in 2021–2022), qualified nurses (76.7% in 2020–2021 vs. 61.5% in 2021–2022), other professionally qualified clinical staff (78.7% in 2020–2021 vs. 63.9% in 2021–2022), and support staff (75.5% in 2020–2021 vs. 56.6% in 2021–2022) [47,48].

The observed coverage is quite similar to the one reached in the year before pandemic. This is against the expected scenario based on the assumption that the COVID-19 pandemic was regarded as an incentive to a good public health practice such as flu vaccination among HCWs [42].

The period of influenza vaccination coincided with the administration of the booster dose against SARS-CoV-2. Although some healthcare workers even opted for the co-administration, many others expressed concerns about the administration within a short period of time, therefore preferring to prioritize the SARS-CoV-2 vaccination and, thus, avoiding the flu one. The main reasons [49] seem to be the fear of the pandemic situation and especially in the uncertainty related to the consequences of a concurrent administration which may overload the immune system or may be more reactogenic, despite the availability of evidence-based recommendations demonstrating its safety and immunogenicity [50]. Moreover, another reason might be the spread of anti-vaccine movements, deriving from the COVID-19 vaccination campaign, even among HCWs, which might also affect the flu vaccination.

Therefore, in order to increase the coverage of influenza vaccination in the next vaccination seasons, it would be advisable to think, in case it would be necessary a booster dose for COVID-19, of a single vaccine to be administered with a single injection as well as to implement timely and innovative organizational approaches.

The differences in vaccine attitudes between physicians and nurses among health care professionals are not only seen in Western countries, such as Italy, but also in East Asian countries, such as Japan. In line with our findings, physicians seem to have a higher willingness to vaccination in Western and Middle East countries [51,52,53,54,55,56]. The reasons underlying this difference might lie in the higher level of knowledge about influenza and influenza vaccines of physicians, the idea of maintaining a strong and healthy body, decisional autonomy, and a perception of untrustworthy environment held by nurses [54,55,56,57].

Besides, according to the evidence in the scientific literature, in Far East countries nurses have a lower vaccine hesitancy compared with physicians. The reason underlying this difference is still debated even though a few scholars have argued that medical doctors do not have enough time to secure vaccination due to their busy schedules [10,58,59].

Before and after the COVID-19 pandemic, the flu vaccination coverage was quite equivalent, implying that, once the acute phase of the emergency gone, HCWs seem to be unwilling to be vaccinated against flu due to the lack of a robust preventive culture. Hence, it is essential to set up and maintain high standards of evidence-based preventive policies (e.g., educational and promotional interventions and on-site vaccinations) [60,61], enhancing them with the lessons learnt from the pandemic experience.

The findings of our report should be assessed considering its limitations and strengths. One caveat is that the findings are based on data from only one hospital which might make the results less representative and biased.

However, our hospital shares common characteristics with other national and international research hospitals, such as a high levels of research activity and a higher internal commitment to guarantee continuous quality improvement so that the care activities are also oriented to innovation assessment and scientific production. Another limitation is the lack of consideration of HCWs vaccinated outside our research hospital; nonetheless, this report is ground-breaking in this field for this flu vaccination campaign (i.e., 2021/22). Finally, an additional limit might be the adoption of a simple linear regression model for fitting data. However, the statistical analysis was conducted according to the proper methodological frameworks and the evidence available in the scientific literature [45,46].

Further studies are required to explore the reasons for health workers’ vaccine hesitancy and to carry out a more incisive information campaign. Additional research, based on well-defined qualitative methods such as surveys, is needed to explore other factors influencing attitudes towards vaccination among HCWs.

It is of paramount importance to not undervalue this red flag. The COVID-19 pandemic represents an opportunity to promote and support large-scale influenza vaccination among HCWs through structured programs, adequate funding [62], and tailored, clear, and consistent communication strategies [63].

## Figures and Tables

**Table 1:** Vaccination coverage by occupation in the last two years.

Occupation	2020/2021		2021/2022		*p*-Value
	Vaccinated (%)	Total	Vaccinated (%)	Total	
Physicians	819 (75)	1089	550 (38)	1461	<0.001
Nurses	970 (48)	2019	455 (20)	2281	<0.001
Other health workers	881 (55)	1603	264 (25)	1337	<0.001
Resident doctors	687 (56)	1229	471 (24)	1969	<0.001
Total health workers	2556 (54)	4685	1740 (25)	7048	<0.001
Administrative staff	666 (54)	1232	239 (20)	1173	<0.001

Note: statistical level was set at *p* < 0.05.

## Data Availability

Data presented in this study are available upon request from the corresponding author.
